# 

**Published:** 1848

**Authors:** 


### CONTENTS.

### PART I.—ORIGINAL COMMUNICATIONS.

ART. 1. Purulent Ophthalmia. By Dr. E. G. Meek. 95
ART. 2. Medical Literature. By G. N Fitch, M.D., Professor in Rush Medical College. 102
ART. 3. Old Dislocation of the Humerus. By H. S. Huber, M.D. 104
ART. 4. A case of Purpurea Hemorrhagica. By W. Wadsworth, M.D. 107
ART. 5. Death following Vaccination. By Dr. P. Gregg. 109
ART. 6. Treatment of Hydrocele. By L. Dunlap, M.D. 110
ART. 7. Case of Abnormal Position of the Intesties, &c., translated from the German of Wolshofer. By Daniel Stahl, M.D. 114
ART. 8. Observations on Cholera Infantum. By J. H. McNutt, M.D. 116
ART. 9. Proceedings of Medical Societies. 121

### PART II.—REVIEWS.

ART. 1. A Practical Treatise on the Causes, Symptoms, and Treatment of Spermatorrhœa. By M. Lallemand, &c. 126

### PART III.—BIBLIOGRAPHICAL NOTICES.

ART. 1. An Introductory Lecture delivered at the Massachusetts Medical College. By Oliver Wendall Holmes, M.D., Professor, &c. 133
ART. 2. The Human Brain; its Structure, Physiology, and Diseases, &c. By Samuel Solly, F. R. S. 143
ART. 3. Theory and Practice of Midwifery. By Fleetwood Churchhill, M.D.; M. R. I. A. &c. 144
ART. 4. The Young Stethoscopist, &c. By Henry J. Bowditch, M.D. 144
ART. 5. Principles and Practice of Surgery. By the late Geo. McClellan, M.D. 145
ART. 6. The Obstetrical Remembrancer, or Denman's Aphorisms, &c. Augmented by Michael Ryan, M.D. 146

### PART IV.—SELECTIONS.

ART. 1. A New Method of Rapidly Uniting Wounds by First Intention. By. S L. Bigelow. 147
ART. 2. On Chloroform. By Professors Simpson and Meigs. 151
ART. 3. Interesting Cases. By James Blake, M.D. 158
ART. 4. On the Theory of Spasmo-Paralysis in Infants and Adults. By Marshall Hall, M.D.; F. R. S. 160
ART. 5. On Restoration to Life. By Robert Brandon, Esq, M. R. C. S. 164
ART. 6. Asclepias Tuberosa. By T. L. Lockwood. 166
ART. 7. On the treatment of Phthisis Pulmonalis. By Dr Hughs Bennett. 167
ART. 8. Poisons and their Antidotes. 169
ART. 9. Mode of Preventing Bed Sores. 176
ART. 10. Influence of Chloroform on the Blood. 177
ART. 11. Effect of Ether and Chloroform on Animal Temperature. 178
ART. 12. New Medical Doctrine. 179

### PART V.—EDITORIAL.

ART. 1. First Annual Meeting of the American Medical Association. 180
ART. 2. Peoria District Medical Society. 183
ART. 3. Importation of Adulterated Drugs and Medicines. 184
ART. 4. Graduates of Rush Medical College. 185
ART. 5. Miscellaneous Medical Intelligence. 186

